# Describing the Challenges of Prehospital Rapid Sequence Intubation by Macintosh Blade Video Laryngoscopy Recordings

**DOI:** 10.1017/S1049023X22000851

**Published:** 2022-08

**Authors:** Clare Hayes-Bradley, Hugo Gemal, Matthew Miller, Sandra Ware

**Affiliations:** 1. PhD Candidate, Department of Paramedicine, SPAHC, University of Monash; Staff Specialist, Prehospital & Retrieval Medicine, Sydney HEMS, NSW Ambulance; Aeromedical Retrieval, New South Wales Ambulance, NSW, Australia; 2. Registrar Prehospital & Retrieval Medicine, Sydney HEMS, NSW Ambulance, NSW, Australia; 3. Staff Specialist, Prehospital & Retrieval Medicine, Sydney HEMS, NSW Ambulance, NSW, Australia; 4. Statistician, Aeromedical Retrieval, NSW Ambulance, NSW, Australia

**Keywords:** intubation, prehospital, video laryngoscopy

## Abstract

**Study Objective:**

Structured review of video laryngoscopy recordings from physician team prehospital rapid sequence intubations (RSIs) may provide new insights into why prehospital intubations are difficult. The aim was to use laryngoscope video recordings to give information on timings, observed features of the airway, laryngoscopy technique, and laryngoscope performance. This was to both describe prehospital airways and to investigate which factors were associated with increased time taken to intubate.

**Methods:**

Sydney Helicopter Emergency Medical Service (HEMS; the aeromedical wing of New South Wales Ambulance, Australia) has a database recording all intubations. The database comprises free-text case detail, airway dataset, scanned case sheet, and uploaded laryngoscope video. The teams of critical care paramedic and doctor use protocol-led intubations with a C-MAC Macintosh size four laryngoscope and intubation adjunct. First-pass intubation rate is approximately 97%. Available video recordings and their database entries were retrospectively analyzed for pre-specified qualitative and quantitative factors.

**Results:**

Prehospital RSI video recordings were available for 385 cases from January 2018 through July 2020. Timings revealed a median of 58 seconds of apnea from laryngoscope entering mouth to ventilations. Median time to intubate (laryngoscope passing lips until tracheal tube inserted) was 35 seconds, interquartile range 28-46 seconds. Suction was required prior to intubation in 29% of prehospital RSIs. Fogging of the camera lens at time of laryngoscopy occurred in 28%. Logistic regression revealed longer time to intubate was associated with airway soiling, Cormack-Lehane Grade 2 or 3, multiple bougie passes, or change of bougie.

**Conclusion:**

Video recordings averaging 35 seconds for first-pass success prehospital RSI with an adjunct give bed-side “definitions of difficulty” of 30 seconds for no glottic view, 45 seconds for no bougie placement, and 60 seconds for no endotracheal tube placement. Awareness of apnea duration can help guide decision making for oxygenation. All emergency intubators need to be cognizant of the need for suctioning. Improving the management of bloodied airways and bougie usage may reduce laryngoscopy duration and be a focus for training. Video screen fogging and missed recordings from some patients may be something manufacturers can address in the future.

## Introduction

Prehospital intubation is known to be challenging. Database studies internationally have investigated factors associated with this difficulty.^1–3^ These reviews of airway registries of intubations performed with direct laryngoscopy Macintosh blades have identified factors such as expertise of operator, blood and/or vomitus in the airway, restricted jaw or neck movement, limited surrounding space, and in some studies, facial trauma, short neck, and obesity as associated with failed first-pass intubation and Cormack-Lehane Grade 3-4.

With the introduction of video laryngoscopy, many services have seen their first-pass rates improve.^4,5^ First-pass success is desirable as it is associated with decreased complications in the critically ill.^6^ It is not known what airway factors are associated with difficult prehospital intubation with video laryngoscopy. If it is known why and how prehospital intubations are challenging, training and protocols can be tailored to address these challenges.

Video laryngoscopy recordings provide new perspectives on the challenges in prehospital intubation. Recordings can show timing of events and record the actual airway encountered by the laryngoscopist. This is an advantage to databases reliant on clinician memory alone. Whether duration of laryngoscopy attempt is important to patient outcome is unknown. Duration of laryngoscopy measured from video laryngoscopy recordings was used as a marker of difficulty.

The aim was to investigate the relationship between duration of laryngoscopy and airway challenges identified from both video and airway database. The hypothesis was that airway anatomy and pathology, laryngoscopy technique, and laryngoscope function could all impact duration of laryngoscopy. Reasons behind longer intubations and a time dependent definition of difficult intubation are proposed.

## Methods

Ethical approval [6457- 2020/ETH00981] for this retrospective observational analysis of routinely collected data was granted by Western Sydney Local Health District Human Research Ethics Committee (New South Wales, Australia). This manuscript follows STROBE guidelines.

### Study Setting

Sydney Helicopter Emergency Medical Service (HEMS; the aeromedical wing of New South Wales Ambulance; Rozelle, New South Wales, Australia) performs approximately 260-300 prehospital rapid sequence intubations (RSIs) per year, predominantly for traumatic injury, with a 97% first-pass success rate. Operating from three bases in New South Wales, Australia, critical care doctor and paramedic teams are dispatched by road, helicopter, and fixed wing, attending approximately 3,000 cases each year. The doctors are consultants or senior trainees from anesthesia, emergency medicine, and intensive care.

The Sydney HEMS clinical practice standard for prehospital RSI^7^ advocates a team approach to optimize first-pass success. This process and checklist are rehearsed at three monthly currencies. “30-second drills” are practiced when encountering difficulty with laryngoscopy. These cover re-checking patient positioning, use of external laryngeal manipulation or occipital lift, suctioning, attempting direct lift of epiglottis with blade, use of video laryngoscopy view, and a “deep midline lift and withdraw” technique before changing operator. “30-second drills” are so named because they can be accomplished within 30 seconds.^7^ The Sydney HEMS preferred technique is to use direct laryngoscopy with video screen confirmation by assistant, with the availability of video laryngoscopy at team discretion. The protocol allows for clinician discretion in whether a patient is preoxygenated (denitrogenated) by spontaneous breathing or assisted breaths. Oxygenation is via bag-valve-mask (BVM) fitted with variable PEEP valve held tightly against the face and nasal cannula. The protocol recommends assisting ventilations and the physician performing intubation when saturations do not reach 98% with this preoxygenation.^7^ All intubations are reviewed in quality assurance activities.

The C-MAC Pocket Monitor devices (Karl Storz; Germany) with reusable metal Macintosh size four blades were introduced at the end of 2017. All teams are encouraged to try to video their laryngoscopies.

The service carries two bougies, a blue hollow Frova intubating catheter (Cook Medical; Denmark) and a white solid plastic bougie (Gum Elastic Bougies – White; Koala Medical; Warriewood, NSW). During the course of this study, the Frova bougie changed from packaged straight to manufactured curved.

After a mission, the physician completes a cloud-based database (Air Maestro V 3.5.20356.1; Avinet Pty. Ltd.; Australia). It is a combination of free-text entry fields, selection boxes, and a detailed airway registry. Uploading of hand-written clinical notes and CMAC videos is expected. Further detail is available on the Sydney HEMS website.

### Selection of Participants

Video laryngoscopy recordings were identified from the database from January 2018 until end of July 2020. Inclusion criteria were RSI (defined as an intubation with muscle relaxant in a patient with signs of life) in a prehospital setting.

### Measurements

Key timepoints for video laryngoscope recordings were decided on before commencement of study, timed to the nearest second. Time zero was taken as laryngoscope passed between lips. Key timepoints were: optimized view of larynx accepted for placing bougie, bougie first seen on screen, bougie passing cords, tracheal tube passing cords, cuff inflation, and ventilations seen on the video as fogging in tracheal tube. Timings were taken from video laryngoscope recordings and not cross referenced with capnograph waveform, although successful intubation is judged by both visual confirmation of placement and end tidal carbon dioxide. In the event of an interrupted attempt, time to laryngoscope passing lips upon removal was measured. Time to intubation was therefore time from laryngoscope passing lips to tracheal tube passing glottis and was selected for the main analysis.

Descriptive factors noted from the videos were: laryngoscopy blade initially inserted into vallecula or lifting epiglottis; airway soiling with blood; suctioning required; grossly abnormal airway anatomy; which bougie or stylet used, number of bougie attempts; fogging of camera lens; video screen “whiteout” from white bougie; and presence of foreign bodies.

To determine if the video cohort were representative of the entire service prehospital RSI practice, comparison was made between the cases with videos and the overall caseload for that period from Air Maestro.

### Data Analysis

Data from Air Maestro were extracted by two retrieval physicians. Data to be abstracted were defined before data collection commenced. In addition to the timings and observations described above, patient detail and airway registry detail were also taken from the database.

Database information used for the analysis were patient age; night time; difficult airway predictor tick boxes (limited mouth opening, cervical spine precautions, obesity, blood or vomit in airway, and facial or neck trauma); intubated by doctor or critical care paramedic; grade of intubation; percentage of glottic opening; critical physiology before RSI drugs given (oxygen saturations <80%, heart rate <50 or >150bpm, systolic blood pressure <80mmHg, and Glasgow Coma Score [GCS] <8 were chosen as variables likely to be consistent with increased stress for the laryngoscopist).

The data collection form was trialed and adjusted before use by both extractors (HG & CHB). For any discrepancies, collective agreement was reached. To confirm interrater reliability and consistency of data, collection was confirmed by funnel plot of variables. Database and chart review was conducted in accordance with good practice.^8,9^

Data were collected on RedCap software (RedCap; Vanderbilt University; USA). Exploratory analysis was performed using statistical computing software (R; R Foundation for Statistical Computing; Vienna). Descriptive statistics utilized parametric and non-parametric tests as appropriate. P <.05 was defined as statistical significance.

A regression analysis was planned to investigate associations between airway factors and time to intubation. First-pass intubations time to intubate was selected as the outcome variable. Factors from both the airway registry and video analysis were then entered as independent variables as identified by relevant existing literature and clinical experience. As this was an exploratory analysis, a stepwise process was planned. Final model residuals would be examined for normality using the Shapiro-Wilk test and qq plot, and for heteroscedasticity using the Breusch-Pagan test to satisfy the assumptions and hence appropriateness of a linear regression before proceeding.

Cases with missing variables for any of the airway factors examined have to be excluded from a linear regression (eg, if the grade of laryngoscopy was not recorded in the database).

## Results

### Characteristics of the Study Subjects

**Figure 1.**
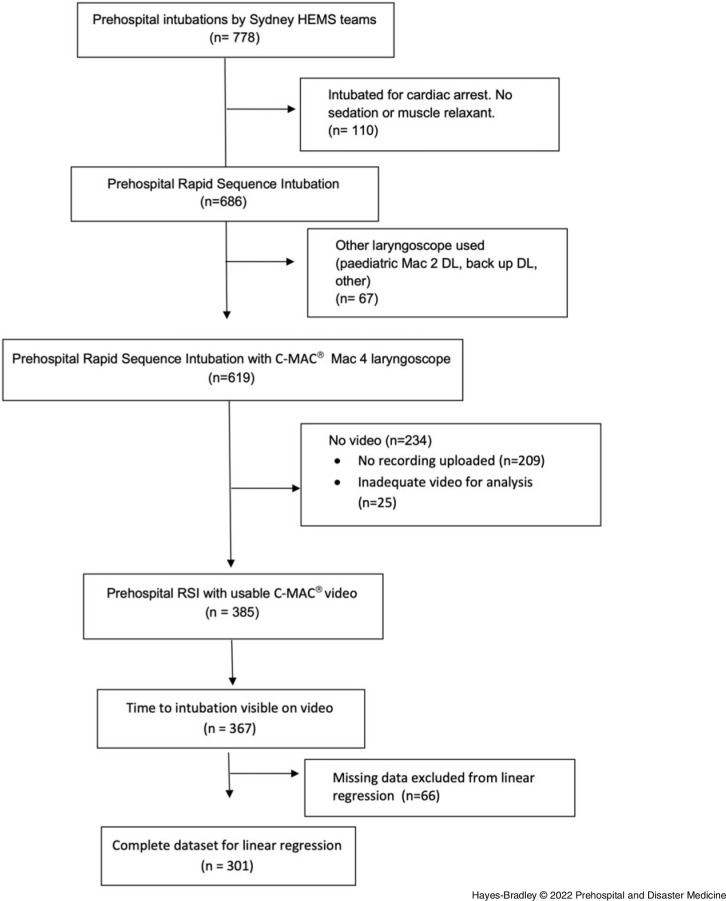
CONSORT Diagram of Identification of Video Laryngoscopy Recordings from Air Maestro Database. Abbreviations: HEMS, Helicopter Emergency Medical Services; DL, direct laryngoscopy; RSI, rapid sequence intubation.

A total of 385 videos of prehospital RSIs were uploaded on the database (Figure 1). The group of 385 intubations with video recordings had similar first-pass success and grade of view to the 234 intubations without videos in the same period (Table 1). Laryngoscopy was Grade 1 or 2 as judged by the clinician in 365 (95%) cases.

**Table 1.** Characteristics of Prehospital Rapid Sequence Intubations from January 2018 – July 2020

	**Video in Database** **(n = 385)**	**No Video in Database** **(n = 234)**
Age (Years)	36 (24-56)	41 (24-58)
Gender: Male	299 (79%)	184 (79%)
Number of Intubation Attempts		
1	373 (97%)	227 (97%)
2	11 (3%)	5 (1%)
3	1 (0.3%)	2 (1%)
>3	0 (0%)	0 (0%)
Clinician Performing Initial Laryngoscopy^a^		
Critical Care Paramedic	277 (74%)	172 (73%)
Critical Care Physician	101 (23%)	62 (28%)
First Attempt C-L Grade Recorded in Database^b^		
1	261 (68%)	155 (66%)
2	104 (27%)	64 (27%)
3	16 (4%)	10 (4%)
4	3 (1%)	3 (1%)

Note: Comparison is made with those C-MAC intubations for which no recording was available to assess ability to extrapolate findings. Values are number (%). Age median (IQR). C-L = Cormack-Lehane laryngoscopy grade.

a Intubator not recorded in eight cases.

b C-L grade not recorded in three cases.

### Time Taken to Intubate

**Figure 2.**
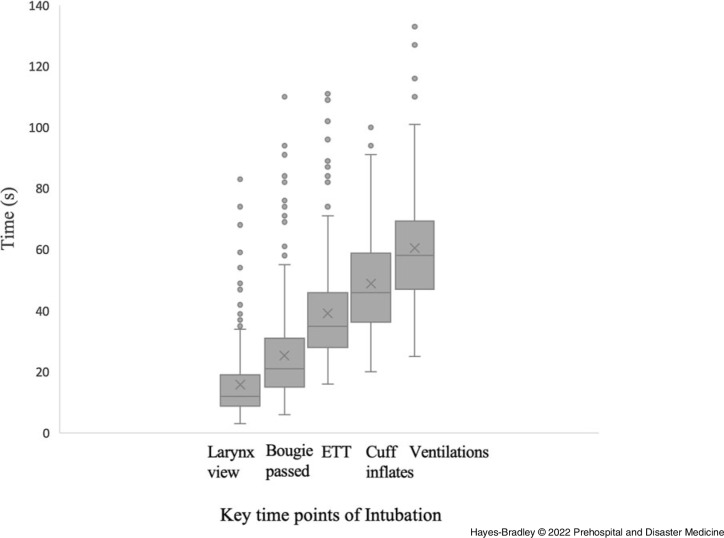
Box and Whisker Plot of Key Timepoints from 373 Successful First Attempt Intubations. Note: Median and interquartile range with dot outliers, values in seconds. X gives mean value (seconds). Abbreviation: ETT, endotracheal tube.

Figure 2 and Table 2 give the key timepoints of the intubations and the number of videos for which those times were available. Missing data were due to failure to visualize glottis or event, video terminating early, screen obscured, or glottis not within screen.

**Table 2.** Key Time Points from Video Recordings of the First-Pass Success Intubation Attempts Giving Number of Videos from which Timings were Assessed.

Key Time Points	Time (Seconds)	No. Videos Showing Time Point
Laryngoscope Placed Between Lips	0	385
Laryngeal View	12 (9-20 [3-83])	371
Bougie Passes Cords	21 (15-31 [6-110])	363
Tracheal Tube Passes Cords	35 (28-46 [16-111])	367
Cuff Inflation Visualized	46 (36-58 [2-101])	92
Ventilations Seen as Fogging in Tracheal Tube (End of Apnea due to Laryngoscopy)	58 (47-69 [17-91])	134
Laryngoscope Passes Lips as Intubation Attempt Abandoned	63.5 (57-84 [17-91])	12

Note: Values are median (IQR [range]). Time to nearest second.

Second or subsequent laryngoscopy was required in 12 cases (3.1 %). First attempt was abandoned at 63 seconds (57-84) median (IQR). Reasons were given in medical notes for interrupting 11 of the 12 abandoned attempts and included: inadequate relaxation (1); desaturation (4); readjusting blade by same operator (2); change of operator (3); and BVM ventilation awaiting smaller tube (1).

### Patient Characteristics and Equipment Challenges

**Figure 3.**
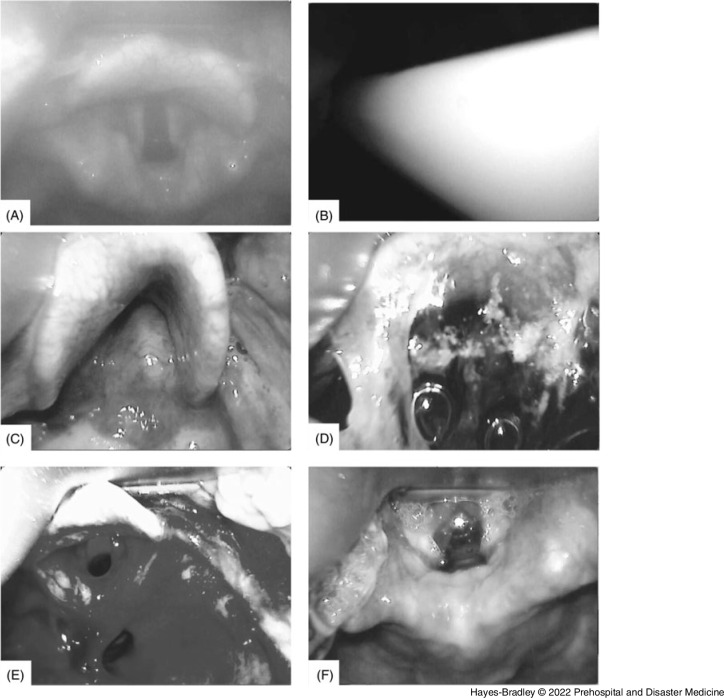
Screenshots from C-MAC Videos Showing Examples of Characteristics of Equipment Performance and Patient Airways. **(A)** Fogging of camera lens. **(B)** Bougie whiteout where arrival of white bougie causes complete loss of laryngeal structures. **(C)** Abnormal anatomy showing enlarged arytenoids/posterior glottic structures. **(D)** Secretions. **(E)** Bloody view. **(F)** Blade tip too close to cords.

The following rates of equipment and patient challenges were noted. In 25% of cases (98/385), the anatomical landmarks were not clearly seen. Suctioning of blood and/or secretions was seen in 113/385 videos (29%). Glottic structures appeared significantly swollen or abnormal in 34 (8.8%) videos and one video revealed a foreign object (examples in Figure 3).

The following equipment challenges were noted. Fogging of the camera lens persisting at the time of view in 108 of 385 videos (28%). The video suffered “whiteout” with a temporary loss of view of glottis during insertion of the white bougie in 12 of 135 cases (9%).

The time between the bougie appearing on the video and it passing the cords was a median (IQR [range]) of two (1-5 [0-70]) seconds. For 10% of first-pass intubations, the bougie was being manipulated on screen for over 15 seconds. The number of bougie passes towards the cords seen during this time was median two (IQR 2-3) but with a range of two to eleven.

### Analysis of Time to Intubate

A small number of outlier observations (n = 26) were omitted from the final model which did not change the regression results. Time to intubate was median 35 seconds with interquartile range 28-46 seconds with range 16 and 111 seconds, as given in Table 2. Outliers (71-89 seconds; n = 8) and extreme outliers (102-111 seconds; n = 5) or those having high leverage on analysis due to extreme independent variables (n = 14) were excluded. As an example, one of the outliers was a case of a suction unit malfunction which had required the sourcing of a second suction unit during the laryngoscopy resulting in a very extended intubation time. As these data were not influential (sensitivity analysis available on request), no bias was introduced by their removal. However, the amount of variance explained by the model was slightly reduced (53% to 47%). This gave 301 complete datasets for the linear regression analysis.

Table 3 shows the results of the linear (multivariate) analysis of airway factors associated with time to intubation. The range of variables used for the regression, described above, was both the airway information in the video recordings defined a priori by the researchers from previous clinical governance experience of watching laryngoscopy videos, and airway data defined a priori by it being collected in the airway registry dataset.

**Table 3.** Multivariate Analysis of Linear Model of Airway Factors Associated with Time to Intubation.

	β	SE β	P Value
(Intercept)	13.54	2.00	
Time to Laryngeal View	0.31	0.05	< .0001
Bougie Passes >1	6.27	0.69	< .0001
Cormack Lehane Grade 2	4.51	1.26	< .0001
Cormack Lehane Grade 3	17.13	5.85	< .001
White Bougie	-3.15	1.04	< .001
Both Bougies Used Sequentially	17.28	8.57	< .01
Larynx Soiled on Video	3.15	1.21	< .001
Blade Tip Adjusted to Pick Up Epiglottis	8.21	2.91	< .001

Note: Only statistically significant results shown here. Influential observations removed to resolve heteroscedasticity. Time in seconds. Adjusted R^2^ 0.47. Reference values single bougie pass, blue Frova introducing catheter bougie, unsoiled glottis, and blade tip introduced into vallecula.

Checkbox criteria for difficult airway predictors identified by the team before laryngoscopy were indicated for 201 of 367 intubations (54.8%), as shown in Table 4. These were incorporated in the linear regression but were not significantly associated with time to intubate.

**Table 4.** Airway Registry Check Boxes for “Difficult Airway Predictors”

Difficult Airway Predictors in Database	385 Cases Included in Video Analysis
Limited Mouth Opening	9 (2.3)
C Spine Precautions	161 (41.8)
Obesity	34 (8.8)
Blood/Vomitus in Airway	86 (22.3)
Facial/Neck Trauma	54 (14.0)
Neck Extension Limited by Anatomy	5 (1.3)

Note: Selected by the reporting physician reflecting patient assessment before intubation for the 385 cases. At least one indicator was selected in 201 of 385 cases (52.2%). n (%). None of these predictors were shown to be associated with increased time to intubate by linear regression.

## Discussion

The average time from start of laryngoscopy until ventilations seen was 58 seconds. Many practitioners were not aware that laryngoscopy and intubation takes one minute on average. This could guide decision making on oxygenation. The choice to hand ventilate before laryngoscopy may be based on the perceived risk of desaturation – which needs to take into account both the time taken waiting for paralysis before laryngoscopy and the further minute for laryngoscopy before ventilations occur.

From the videos, the following definitions of difficult intubation for prehospital systems utilizing CMAC Macintosh laryngoscopes with bougies is proposed. Difficult laryngoscopy would be a view of the glottis not achieved within 30 seconds (32 of 371 videos; 8.6%). Difficult bougie placement would be not passed by 45 seconds (36 of 363 videos; 9.9%). Difficult intubation would be those taking longer than 60 seconds for tracheal tube to pass cord (33 of 368 [9.0%] took longer than 60 seconds). Teams use “30-second drills”^7^ to troubleshoot difficult views, so named because they can be completed within 30 seconds. This study suggests they be started at 30 seconds also.

The soiled airway is well-known to prehospital practitioners^3,4^ and is reflected in this dataset where one in three cases needed suctioning. Together with screen fogging and bougie whiteout, the risk of camera soiling highlights the need for maintaining skills in direct laryngoscopy for prehospital providers. Training for airway soiling events seems warranted.

The need for multiple bougie passes may reflect a challenging laryngoscopy, room for skill improvement, or a combination. Within this cohort of 97% first-pass intubations, 10% of those intubations took longer than 15 seconds to place a bougie after gaining a view of the glottis. Whether changing bougie equipment, its storage, or bougie skills training could reduce the number of bougie passes remains unknown.

None of the bed-side difficult airway predictors from the airway registry (historically associated with multiple attempts to intubate) showed as a relationship with first-pass time to intubate. Whilst it is tempting the dismiss these historical predictors as not useful, it may be that the approach used effectively mitigates for these well-known factors.

## Limitations

Only 385 videos from 694 prehospital RSI intubations were studied, although the characteristics of the video group marry those of the overall workload. Challenges to videoing are both inherent in the device and human in nature. Hopefully technology will provide some solutions in the future.

Statistical analysis dependent on assumptions of the linear model often requires certain compromises, such as excluding extreme outliers. This reduces the number of cases available to the analysis. Fortunately, sensitivity analysis showed that the removal of outlier values did not alter the results and conclusions from the regression.

The C-MAC images are only one small part appreciating the challenges in prehospital intubation. The image does not capture the entire pharyngeal space. There are of course challenges outside of the mouth – eyewear fogging, reduced manual dexterity in the cold, and equipment failures, to name a few. The video recordings are a valuable part of quality assurance process and can be seen on the Sydney HEMS website.^10^

Future work could investigate the relationship with patient outcomes such as desaturation, regurgitation, or airway trauma for prolonged attempts, and whether they are desirable to multiple attempts.

## Conclusions

Knowledge of average timings could aid decision making on value of continuing or interrupting a laryngoscopy attempt. Linear regression identified a soiled airway and multiple attempts to pass a bougie are associated with increased time to intubate. In analyzing timings, it is impossible to distinguish between operator, equipment, context, and patient and one should be cautious in interpretation. Bloody airways and bougie technique would be a worthwhile focus for future training. The CMAC screen fogging and difficulty recording are areas for future technological advance.
